# Symmetrical Peripheral Gangrene: Report of Three Cases

**DOI:** 10.1155/2022/8615420

**Published:** 2022-10-12

**Authors:** Héctor Acosta, Pau Forcada, Marta Bonjorn, Amer Mustafa, Paul Pilares, Jordi Colomina

**Affiliations:** ^1^Hospital Universitari de Santa Maria, 25198 Lleida, Spain; ^2^Hospital Universitari Arnau de Vilanova, 25198 Lleida, Spain

## Abstract

Symmetrical peripheral gangrene (SPG) is a rare clinical syndrome characterized by an acute onset of ischemic damage in two or more extremities without obstruction or vasculitis of supplying vessels. Body parts commonly affected include toes, hands, scrotum, and earlobes, increasing the risk of limb amputation and impairing the quality of life. The vascular injury mechanism is disseminated intravascular coagulation. SPG may manifest unpredictably in conditions associated with septic shock, low output states, vasospastic conditions, myeloproliferative disorders, or hyperviscosity syndrome. A review on the topic is presented based on a 3-case series of SPG that required amputation of fingers and toes after the administration of vasopressors in a septic shock context.

## 1. Introduction

Symmetrical peripheral gangrene (SPG) is a rare condition in which symmetrical ischemia and dry gangrene of the acral areas occur. Body parts commonly affected include the toes, hands, scrotum, and earlobes, increasing the risk of limb amputation and affecting the quality of life [1].

The aetiology is multifactorial but has been linked to vasopressor's use during the treatment of septic shock [1, 2]. There is consensus in the literature that early recognition of PSG, along with underlying conditions, can change significantly the treatment and the final evolution [2].

The vascular injury mechanism is disseminated intravascular coagulation (DIC) [3, 4]. Several infectious and noninfectious etiologic factors have been associated with SPG [2, 5–7]. It has been described in conditions associated with sepsis, low blood flow states, vasospastic conditions, hyperviscosity disorders, and myeloproliferative disorders [8]. The appearance of SPG related to the inappropriate use of vasopressor drugs also has been described [9].

Dopamine and norepinephrine are the first-choice drugs in septic shock cases due to their positive inotropic effects. SPG can occur with prolonged administration, especially at high infusion rates. SPG can cause catastrophic complications with high mortality rates and high frequencies of multiple limb amputations in up to 70% of surviving patients [8, 9]. We present the case of a patient with SPG associated with vasopressor use who required amputation of all fingers and toes.

## 2. Case Series

### 2.1. Case 1

A 46-year-old woman with history of uterine fibroids after elective hysterectomy presented with surgical wound infection evolving to septic shock that required admission to the intensive care unit (ICU).

During admission, the patient developed multiorgan failure, abdominal compartment syndrome, and DIC. Positive cultures for *Pseudomonas aeruginosa*, *Enterococcus faecalis*, and *Candida tropicalis* were obtained, for which intravenous antibiotics and antifungals were administered. Due to the hemodynamic instability, it was necessary to administer vasopressors to maintain systolic blood pressure and mean arterial pressure goals.

**Figure 1:**
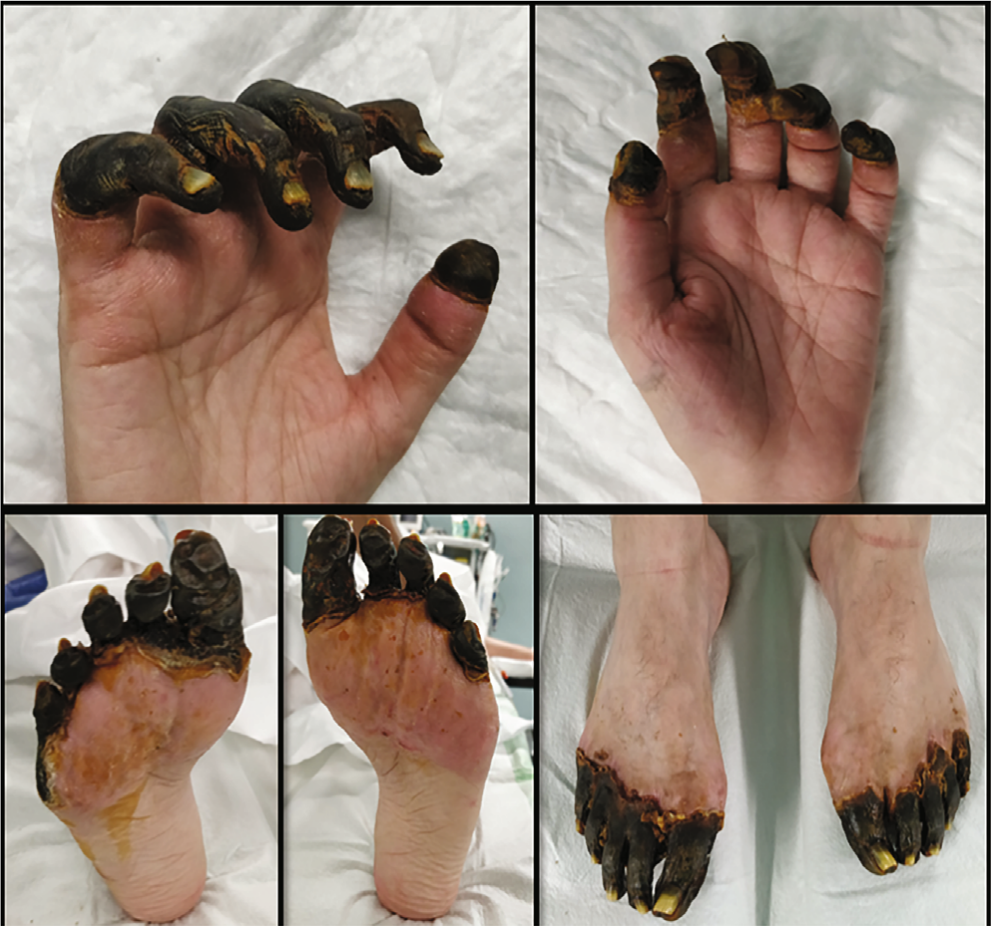
Case 1 lesions.

With increasing doses of 1.5 *μ*g/kg/min of norepinephrine, the patient's lactate was 6.6 mmol/L on the same day. On the third day of admission to the ICU, the patient underwent surgery again due to suspected abdominal compartment syndrome; twenty-four hours later, the norepinephrine dose was decreased, and a lower lactate level of 4 mmol/L was observed. On the fifth day of ICU admission, norepinephrine was withdrawn, and that same day, the presence of greyish lesions was observed on both hands, finger pads that evolved into haemorrhagic blisters on the left hand and patchy purple lesions on both feet and toes and stayed cyanosis in the heels. Radial, ulnar, pedal, and posterior tibial pulses of all extremities were normal on Doppler examination. The lesions evolved into necrotic areas at the metacarpal and phalangeal levels in both hands and distal phalangeal levels in both feet (Figure 1).

The patient received ventilatory support treatment, dialysis, and antithrombotic treatment with low molecular weight heparin, remaining in the ICU for 54 days. During necrotic lesions' delimiting time, wound care was performed with dry dressings and limbs covered with padded bandages.

**Figure 2:**
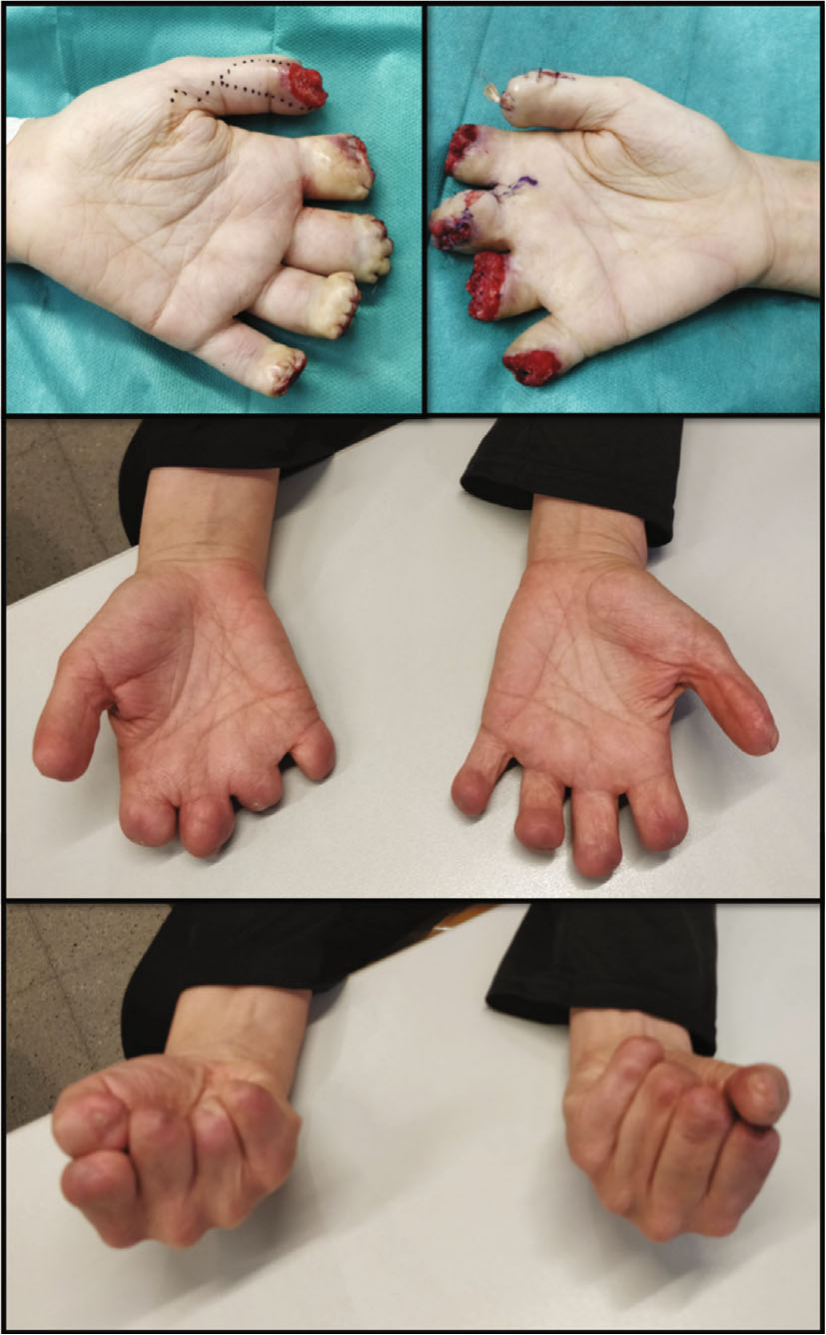
Intraoperative levels of amputation and functionality after complete wound healing.

In gangrenous areas, surgical amputations were performed. In the right hand, the four triphalangeal finger stumps were regularized at F1 transphalangeal level, and bone coverage with deep plane and closure by second intention was performed. In the 3rd finger, a radial pedicled flap advancement was performed, and in the thumb, regularization of the second phalanx and coverage with a Moberg-type flap was performed, leaving the matrix and part of the nail bed. Transphalangeal amputation of the 2nd and 3rd fingers was performed on the left hand associated with deep plane coverage, fingers 4th and 5th were amputated at the level of the middle phalanx, and radial flap advancement was performed on the thumb to close the cutaneous defect, maintaining part of the matrix and the nail bed. Despite the aggressiveness of the amputation surgery, the patient retains a certain ability to grip both hands and an acceptable prehension of the thumb and index on the left hand. Result of the surgeries performed can be seen in Figure 2.

Transmetatarsal amputation of the second to the fifth finger and proximal transphalangeal amputation of the first finger were performed on the left foot. On the right foot, she required complete transmetatarsal amputation plus posterior grafting.

One year later, she continued her recovery as an outpatient, presenting with right thumb nail matrix discomfort, requiring matrix surgery and sterilization, with no reports of other complications.

### 2.2. Case 2

A 53-year-old man was in ICU after septic shock secondary to appendicitis that progressed rapidly to peritonitis and multiple organ failure with DIC.

**Figure 3:**
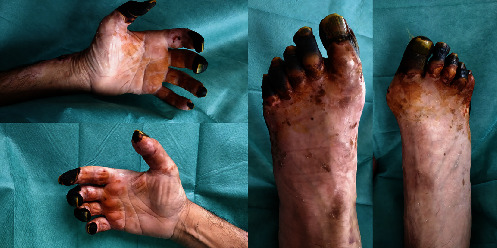
Case 2 lesions.

During admission, norepinephrine administration was necessary to maintain hemodynamic stability. The patient underwent an urgent laparotomy and negative pressure therapy. Positive cultures for *E. coli* were obtained, which received targeted antibiotic treatment. Initial lactate was 6.8 mmol/L on the first day of admission. On the fourth day of admission, he presented ischemic skin lesions on the fingers of both hands and feet toes. On the left hand, nonsuppurative, dry, and well-defined necrosis stablished at the distal level of the fingers 1st, 2nd, and 3rd with proximal interphalangeal joint involvement, 4th and 5th were only affected in the pad, and also, a superficial necrotic eschar at the left dorsal hand level and wrist on its radial face appeared. On the right hand, well-defined nonsuppurative necrosis was observed in the distal ulnar aspect of the thumb with partial involvement of the finger pad, necrosis 2nd, 3rd, and 4th fingers with involvement up to PIP, finger 5 with involvement of the finger pad. Both feet presented poorly defined dry necrosis affecting all toes of both feet. For images of the described lesions, see Figure 3.

The patient received ventilatory support treatment, dialysis, and antithrombotic treatment with low molecular weight heparin, remaining in the ICU for 42 days. During the delimiting necrotic lesion time, wound care was performed with dry dressings and limbs covered with padded bandages.

The patient died on day 42.

### 2.3. Case 3

A 43-year-old woman with a history of acute myeloid leukaemia (AML) presented with a septic shock of abdominal origin caused by colitis, requiring admission to the ICU due to multiple organ failure and DIC.

She received treatment with norepinephrine to maintain mean blood pressure above 65 mmHg. Klebsiella and Pseudomonas were identified in the cultures, for which specific antibiotic treatment was given. Initial lactate was 11.2 mmol/L, decreasing to 1.2 mmol/L after a week, presenting thrombocytopenia for which platelet transfusion was necessary during admission. On the 12th day of admission, she was assessed for traumatology due to skin lesions on both hands with nonsuppurative dry necrosis of the tips of fingers 4th and 5th of the left hand, dry necrosis of the 5th finger of the right hand that affects up to the distal interphalangeal joint (DIP), 4th finger necrosis that affects up to the middle phalanx (F2) without reaching the joint, and 3rd finger and thumb with only of the distal fingertip involvement. There were no involvement of the toes or other acral areas in this case.

She was taken to surgery, where the necrotic tip of the 4th finger of the right hand was removed, with distal phalanx (F3) regularization and removal of the remaining nail matrix, Atasoy-type coverage was performed. In the 5th finger, the necrotic tip was removed, regularized with disarticulation at the DIP joint level and removal of cartilage remains at the distal level of F2. Fish mouth coverage type and wound closure with 5/0 monofilament. Wounds are treated and padded bandage until discharge.

After 22 days of hospitalization due to persistent fever and haemorrhagic complications, the patient presented clinical improvement and was discharged from the hospital. She is recovering on an outpatient basis with good functionality of the hand, for which she was discharged from traumatology after 6 follow-up weeks. No other complications were recorded to date.

Demographic and causal factors of the three cases summarized in Table 1. And clinical presentation and treatments are collected in Table 2.

## 3. Discussion

Symmetrical peripheral gangrene or purpura fulminans [10, 11] is a rare syndrome characterized by an acute onset of ischemic damage in peripheral acral areas without obstruction or vasculitis of the supplying vessels [1, 6].

There are no large studies that provide scientific evidence about this rare condition [8], being in this article 3 cases reported.

Altered microcirculation and vasospastic conditions with poor peripheral perfusion are the main clinical scenarios associated with SPG [1, 6, 9].

Disseminated intravascular coagulation (DIC) could be the last cause of microvascular injury. This association was first described in 1970 by Strossel and Levy [6]. The possibility that SPG may be one special cutaneous variant of DIC has been described [2].

In patients with DIC and hypovolemia, the use of vasopressor drugs like epinephrine, dopamine, and norepinephrine can cause SPG by decreasing tissue perfusion and exacerbating ischemia, leading to eventual tissue necrosis and gangrene [2, 12].

Some studies describe that the dose instead of the duration could be more important in the development of SPG and also have additive effects [2, 9, 12].

In this report, all 3 cases were caused by septic conditions [13] which lead to DIC and precise norepinephrine at curative levels developing SPG in a few days, 4 to 12.

The first suggestive sign is the presence of cold, erythematous extremities followed by dark discoloration of the skin. Ischemic changes progressing to cyanosis and hemorrhagic bullae may develop in symmetric acral distribution over the fingers and toes. In severe cases, involvement of the nose, earlobe, or scrotum can be observed [10]. Of the reported cases, two had both hands and feet involved and the 3rd only hands, no other body parts were involved.

In the first 12-24 hours, dry gangrene appears and progresses proximally with a demarcation line that develops in approximately two or three weeks [6, 8].

Laboratory tests like complete blood count with blood smear help to identify myeloproliferative or infectious alterations. D-dimer can indicate the presence of DIC. Blood biochemistry helps detect chronic pathologies. In patients with septic shock, high levels of serum lactate can be observed just before the appearance of SPG [2, 6]. In all cases, reported serum lactate was elevated from 6.6 to 11.2 mmol/L.

The initial management is hemodynamic stabilization. Early recognition of cyanosis, reversal of DIC, and correction of hypovolemia are essential to prevent the progression to gangrene. Effective treatment of underlying pathologies like antibiotic treatment for sepsis will determine the outcome in individual cases. The use of heparin in the treatment of DIC remains controversial, although it may be useful in patients with clinically evident thromboembolism and extensive fibrin deposition. Antithrombin III alone or in association with heparin appears to be a promising agent [6].

The only definitive treatment established for gangrene of acral areas is amputation. It should be considered after the development of a clear line of demarcation, until this moment emphasis should be placed on local wound care [2]. Also, self-amputation of the gangrenous digits on very distal lesions could be seen [2, 6].

The level of amputation must be decided during surgery. Separate bone and soft tissue reconstruction leads to good results in terms of wound healing and ambulatory status [2]. In most cases of fingers, a V-Y advancement flap can be performed in either volar or a double lateral flap trying to maintain the maximum finger length, especially in thumb and index fingers used for pinch grip. Treatment in our case was amputation using different flap types like Moberg, Atasoy, or fish mouth were performed with acceptable functional results.

For the lower extremities, microsurgical techniques and the use of free flaps have been described with good functional results [14, 15]. In case one, foot amputations were performed using a skin graft in the right foot with no other complications.

## 4. Conclusions

SPG is a rare condition that mainly affects critical patients who require the use of inotropes such as norepinephrine. In these patients, strict surveillance of the acral areas is necessary during treatment to detect ischemic signs in time and correct the already existing DIC since the development of SPG can lead to limb amputation with serious impairment of quality of life. Treatment, once dry gangrene is established, consists of amputation of necrotic areas when they are demarcated trying to preserve functionality if possible. Clinical suspicion and early diagnosis is key improve its management.

## Figures and Tables

**Table 1:** Summary of the demographic and causal factors of the three cases.

Case	Sex age	Primary causal disease	Secondary casual disease	Cultures	Vasopressor	ICU days
1	M 46	Elective hysterectomy	Septic shock	*Enterococcus faecalis, Pseudomonas Aeruginosa*	Norepinephrine	54
2	F 53	Appendicitis	Septic shock	*Escherichia coli*	Norepinephrine	42
3	M 43	Colitis	Septic shock	*Klebsiella pneumoniae*	Norepinephrine	22

**Table 2:** Summary of clinical presentation and treatment of the three cases.

Case	All extremities affectation	Clamp impairment	Days from admission to onset	Treatment
1	Yes	Yes	5	All finger amputation
2	Yes	Yes	4	Death before surgery
3	No, both hands' affectation	Thumb without clamp function impairment	12	Right hand 4, 5 finger amputation

## Data Availability

The data that support the findings of this study are available from the corresponding author, upon reasonable request. Sensible information that could lead to personal patient identification will not be provided.
